# Pretreatment of Lignocellulosic Wastes to Improve Ethanol and Biogas Production: A Review

**DOI:** 10.3390/ijms9091621

**Published:** 2008-09-01

**Authors:** Mohammad J. Taherzadeh, Keikhosro Karimi

**Affiliations:** 1 School of Engineering, University of Borås, 501 90 Borås, Sweden; 2 Department of Chemical Engineering, Isfahan University of Technology, Isfahan, 84156-83111, Iran. E-Mail: Keikhosro.Karimi@hb.se

**Keywords:** Pretreatment, biogas, methane, ethanol, lignocellulose, waste, biodegradation

## Abstract

Lignocelluloses are often a major or sometimes the sole components of different waste streams from various industries, forestry, agriculture and municipalities. Hydrolysis of these materials is the first step for either digestion to biogas (methane) or fermentation to ethanol. However, enzymatic hydrolysis of lignocelluloses with no pretreatment is usually not so effective because of high stability of the materials to enzymatic or bacterial attacks. The present work is dedicated to reviewing the methods that have been studied for pretreatment of lignocellulosic wastes for conversion to ethanol or biogas. Effective parameters in pretreatment of lignocelluloses, such as crystallinity, accessible surface area, and protection by lignin and hemicellulose are described first. Then, several pretreatment methods are discussed and their effects on improvement in ethanol and/or biogas production are described. They include milling, irradiation, microwave, steam explosion, ammonia fiber explosion (AFEX), supercritical CO_2_ and its explosion, alkaline hydrolysis, liquid hot-water pretreatment, organosolv processes, wet oxidation, ozonolysis, dilute-and concentrated-acid hydrolyses, and biological pretreatments.

## 1. Introduction

Production of waste materials is an undeniable part of human society. The wastes are produced by several sectors including industries, forestry, agriculture and municipalities. The accumulation of waste and the “throw-away philosophy” result in several environmental problems, health issues and safety hazards, and prevent sustainable development in terms of resource recovery and recycling of waste materials. A perspective aimed at promoting greater sustainable development and resource recovery has influenced solid waste management practices, and is gradually becoming implemented through policy guidelines at national levels in a number of industrialized and even developing countries. Guidelines and directives to reduce waste generation and promote waste recovery are laid down according to the “waste management hierarchy”, in which waste prevention, reuse, recycling and energy recovery are designed to minimize the amount of waste left for final, safe disposal [1].

Ethanol is now the most important renewable fuel in terms of volume and market value [2]. Nowadays it is produced from sugar-and starch-based materials such as sugarcane and corn. However, the second generation production of ethanol derived from lignocellulosic materials is now being tested in pilot plants [3, 4]. The present work paves the way for integrating waste streams into the raw materials for ethanol plants, by reviewing different methods for their pretreatment for improved production of ethanol.

Biogas is another energy source that is used as car fuel, or for production of heat or electricity in different countries [5, 6]. Biogas production from activated sludge is an old and almost established process. It has also recently been produced on industrial scales from municipal solid waste (MSW) and some homogeneous wastes such as manures. Forestry and agriculture residues and MSW are by nature heterogeneous in size, composition, structure, and properties. Sugars, starches, lipids and proteins present in MSW are among the materials easily degradable by microorganisms, while some other fractions such as lignocelluloses and keratin are more difficult to degrade [7]. Biological degradations of these polymers are carried out by several enzymes such as amylase, cellulase, protease, keratinase and lipase, before further fermentation or digestion to e.g. ethanol or biogas. However, these polymers should be accessible to the enzymes for biodegradation.

**Figure 1.**
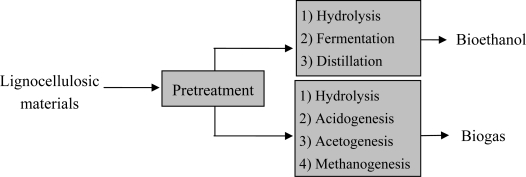
Pretreatment of lignocellulosic materials prior to bioethanol and biogas production

Pretreatment by physical, chemical or biological means is a well-investigated process for ethanol production from lignocellulosic materials. Furthermore, there have been some efforts to pretreat waste materials for biogas production. The pretreatment can enhance the bio-digestibility of the wastes for ethanol and biogas production and increase accessibility of the enzymes to the materials. It results in enrichment of the difficult biodegradable materials, and improves the yield of ethanol or biogas from the wastes (Figure 1).

The present work deals with reviewing the pretreatment processes used in ethanol and biogas processes from waste materials. However, since wastes comprise a wide range of materials, we dedicate this review only to lignocellulosic part of waste materials in order to shorten the discussion.

## 2. Lignocelluloses among waste materials

In addition to inorganic wastes, different types of polymers are available in various waste materials. Natural materials such as starch, lipids, glycogen, elastin, collagen, keratin, chitin and lignocelluloses, as well as synthetic polymers such as polyesters, polyethylene and polypropylene, are among these polymers. In this work, we focus on treatment of lignocellulosic polymers that are resistant to biological degradation.

**Figure 2.**
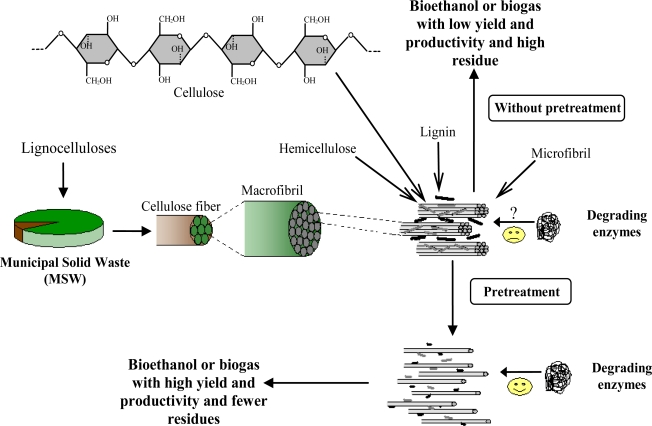
Effect of pretreatment on accessibility of degrading enzymes

Lignocelluloses (Figure 2) comprise a large fraction of municipal solid waste (MSW), crop residues, animal manures, woodlot arisings, forest residues, or dedicated energy crops [5]. Lignocelluloses are composed of cellulose, hemicellulose, lignin, extractives, and several inorganic materials [8]. Cellulose or β-1-4-glucan is a linear polysaccharide polymer of glucose made of cellobiose units [9, 10]. The cellulose chains are packed by hydrogen bonds in so-called ‘elementary and microfibrils’ [11]. These fibrils are attached to each other by hemicelluloses, amorphous polymers of different sugars as well as other polymers such as pectin, and covered by lignin. The microfibrils are often associated in the form of bundles or macrofibrils [9] (Figure 2). This special and complicated structure makes cellulose resistant to both biological and chemical treatments. Cellulose is available in waste streams in the form of lignocelluloses, or partly purified in the form of e.g. papers or pure cellulose such as cotton, or mixed with other materials, in e.g. citrus wastes [12].

The dominant sugars in hemicelluloses are mannose in softwoods and xylose in hardwoods and agriculture residues [13–15]. Furthermore, these heteropolymers contain galactose, glucose, arabinose, and small amounts of rhamnose, glucuronic acid, methyl glucuronic acid, and galacturonic acid. In contrast to cellulose, which is crystalline and strong, hemicelluloses have a random, amorphous, and branched structure with little resistance to hydrolysis, and they are more easily hydrolyzed by acids to their monomer components [8, 10, 16–18].

Lignin is a very complex molecule constructed of phenylpropane units linked in a three-dimensional structure which is particularly difficult to biodegrade. Lignin is the most recalcitrant component of the plant cell wall, and the higher the proportion of lignin, the higher the resistance to chemical and enzymatic degradation. Generally, softwoods contain more lignin than hardwoods and most of the agriculture residues. There are chemical bonds between lignin and hemicellulose and even cellulose [19, 20]. Lignin is one of the drawbacks of using lignocellulosic materials in fermentation, as it makes lignocellulose resistant to chemical and biological degradation.

## 3. Effective parameters in pretreatment of lignocelluloses

The inherent properties of native lignocellulosic materials make them resistant to enzymatic attack. The aim of pretreatment is to change these properties in order to prepare the materials for enzymatic degradation. Since lignocellulosic materials are very complicated, their pretreatment is not simple either. The best method and conditions of pretreatment depend greatly on the type of lignocelluloses. For instance, pretreatment of bark from poplar trees or corn leaf with a dilute-acid process seems to be promising, but this method is not effective for treating the bark from sweetgum or corn stalks [21, 22].

The crystallinity of cellulose, its accessible surface area and protection by lignin and hemicellulose, degree of cellulose polymerization, and degree of acetylation of hemicelluloses are the main factors considered as affecting the rate of biological degradation of lignocelluloses by the enzymes [23]. These factors will be discussed briefly below.

### 3.1. Crystallinity

The cellulose microfibrils have both crystalline and amorphous regions, and the crystallinity is given by the relative amounts of these two regions. The major part of cellulose (around 2/3 of the total cellulose) is in the crystalline form [24]. It was shown that cellulase readily hydrolyzes the more accessible amorphous portion of cellulose, while the enzyme is not so effective in degrading the less accessible crystalline portion. It is therefore expected that high-crystallinity cellulose will be more resistant to enzymatic hydrolysis, and it is widely accepted that decreasing the crystallinity increases the digestibility of lignocelluloses [25].

On contrary, there are some studies [23, 26] that show more digestibility of more crystalline lignocelluloses. This conflict in the reports might appear, while the effects of other factors are ignored. Grethlein [26] pretreated hard-and softwoods by mild acid hydrolysis and determined their pore size distribution. Regardless of the substrate, the initial rate of hydrolysis was shown to be linearly correlated with the pore volume of the substrate accessible to the size of the cellulase. However, it was also shown that the crystallinity index has no relationship to the rate of hydrolysis. Kim and Holtzapple [27] found that the degree of crystallinity of corn stover slightly increased from 43% to 60% through delignification with calcium hydroxide, which was related to removal of amorphous components (lignin, hemicellulose). However, an increase in crystallinity of pretreated material did not negatively affect the yield of enzymatic hydrolysis. Fan *et al*. [25] studied the effect of ball milling on surface area and crystallinity of cellulose. They observed an increase in crystallinity of cellulose by reducing the size of cellulose by milling. It is believed that recrystallization during water swelling may increase the crystallinity of highly ball-milled cellulose.

This discussion may indicate that the crystallinity is an important factor in digestibility of lignocelluloses. However, it is not the only factor in effective enzymatic hydrolysis of these materials, due to the heterogeneous nature of celluloses and the contribution of other components such as lignin and hemicellulose.

### 3.2. Effect of accessible surface area

Several studies have shown a good correlation between the pore volume or population (accessible surface area for cellulase) and the enzymatic digestibility of lignocellulosic materials. The main reason for improvement in enzymatic hydrolysis by removing lignin and hemicellulose is related to the cellulose accessible surface area. The effect of this area may correlate with crystallinity or lignin protection or hemicellulose presentation or all of them. Therefore, many researchers have not considered the accessible surface area as an individual factor that affects the enzymatic hydrolysis [23]. The first part of enzymatic hydrolysis consists of [23, 28]: (I) adsorption of cellulase enzymes from liquid phase onto the surface of cellulose (solid), (II) biodegradation of cellulose to simple sugars, mainly cellobiose and oligomers, and (III) desorption of cellulase to the liquid phase. Thus, the reaction is a heterogeneous catalytic reaction and direct physical contact between the cellulytic enzymes’ molecules and cellulose is a prerequisite for enzymatic hydrolysis. As a result, the accessible surface area in lignocellulosic material and its interaction with the enzymes can be limiting in enzymatic hydrolysis [25, 28, 29].

Lignocellulosic materials have two different types of surface area: external and internal. The external surface area is related to the size and shape of the particles, while the internal surface area depends on the capillary structure of cellulosic fibers. Typically, dry cellulosic fibers have small size, about 15 to 40 μm, and therefore they possess a considerable external specific surface area, e.g. 0.6–1.6 m^2^/g. However, the internal surface area of dried cellulosic fibers is smaller than the external surface area. Swelling of lignocelluloses with water and polar solvents creates a very large internal surface area [25]. Drying of fibers can result in irreversible collapse and shrinking of the capillary and thus reduce the accessible surface area. Presence of water has a significant effect on the specific surface area of natural cellulose. The specific surface area is known to increase with wetting. Water is known to increase the crystallinity of cellulose, due to a re-crystallization of highly amorphous cellulose.

The accessible surface area changes during enzymatic hydrolysis. The rate of hydrolysis is usually very high at first, and then decreases in the later stages. The specific surface area, or accessible surface area per gram of substrate (m^2^/g), sharply increases during the initial stage. However, it was shown that the cellulose surface area is not a major limiting factor for hydrolysis of pure cellulose [25]. In other words, the slowdown of hydrolysis in the later stages is not due to a lack of associable surface area, but to the difficulty in hydrolysis of crystalline part of cellulose. Therefore, one may expect a lower rate of hydrolysis after hydrolysis of the amorphous cellulose [25].

### 3.3. Effect of lignin

The cellulose and hemicellulose are cemented together by lignin. Lignin is responsible for integrity, structural rigidity, and prevention of swelling of lignocelluloses. Thus, lignin content and distribution constitute the most recognized factor which is responsible for recalcitrance of lignocellulosic materials to enzymatic degradation by limiting the enzyme accessibility; therefore the delignification processes can improve the rate and extent of enzymatic hydrolysis. However, in most delignification methods, part of the hemicellulose is also hydrolyzed, and hence the delignification does not show the sole effect of lignin [23]. Dissolved lignin due to e.g. pretreatment of lignocelluloses is also an inhibitor for cellulase, xylanase, and glucosidase. Various cellulases differ in their inhibition by lignin, while the xylanases and glucosidase are less affected by lignin [30].

The composition and distribution of lignin might also be as important as the concentration of lignin. Some softwoods are more recalcitrant than hardwoods. This might be related to the lignin type, since softwoods have mainly guaiacyl lignin while hardwoods have a mix of guaiacyl and syringyl lignin. It has been suggested that guaiacyl lignin restricts fiber swelling and enzyme accessibility more than syringyl lignin [31].

In some investigations (e.g. [32]), the inhibitory role of lignin has been related to its effect on cellulose swelling. On the other hand, the swelling can be achieved without removal of lignin, and it does not increase the pore size or the extent of hydrolysis. However, it was shown that lignin still has a significant effect on enzymatic digestibility, even in cases where it no longer prevents fiber swelling. The reason for improved rate of hydrolysis by removal of lignin might be related to a better surface accessibility for enzymes by increasing the population of pores after removing of lignin.

### 3.4. Effect of hemicellulose

Hemicellulose is a physical barrier which surrounds the cellulose fibers and can protect the cellulose from enzymatic attack. Many pretreatment methods were shown to be able to remove hemicelluloses and consequently improve the enzymatic hydrolysis. But most of these processes partly remove the lignin as well, so the improvement is not the result of removal of hemicellulose alone [23]. The accessible surface for enzymatic attack may be related to cellulose crystallinity, lignin, and hemicellulose content. Hemicellulose can be hydrolyzed by enzymatic hydrolysis by hemicellulase. However, a suitable pretreatment, e.g. dilute-acid treatment which removes the hemicellulose, eliminates or reduces the need for use of hemicellulase enzyme mixtures for degrading of biomass [33].

## 4. Pretreatment methods for lignocellulose wastes

To achieve enzymatic degradation in production of ethanol by enzymatic hydrolysis or in order to improve formation of biogas, a pretreatment process is necessary. An effective and economical pretreatment should meet the following requirements: (a) production of reactive cellulosic fiber for enzymatic attack, (b) avoiding destruction of hemicelluloses and cellulose, (c) avoiding formation of possible inhibitors for hydrolytic enzymes and fermenting microorganisms, (d) minimizing the energy demand, (e) reducing the cost of size reduction for feedstocks, (f) reducing the cost of material for construction of pretreatment reactors, (g) producing less residues, (h) consumption of little or no chemical and using a cheap chemical.

Several methods have been introduced for pretreatment of lignocellulosic materials prior to enzymatic hydrolysis or digestion. These methods are classified into “Physical pretreatment”, “Physico-chemical pretreatment”, “Chemical pretreatment”, and “Biological pretreatment” [23, 30, 34–56]. The methods of pretreatment of lignocellulosic materials are summarized in Table 1. In this section, we review these methods, although not all of them have yet developed enough to be feasible for applications in large-scale processes.

### 4.1. Physical pretreatment

Physical pretreatment can increase the accessible surface area and size of pores, and decrease the crystallinity and degrees of polymerization of cellulose. Different types of physical processes such as milling (e.g. ball milling, two-roll milling, hammer milling, colloid milling, and vibro energy milling) and irradiation (e.g. by gamma rays, electron beam or microwaves) can be used to improve the enzymatic hydrolysis or biodegradability of lignocellulosic waste materials.

#### 4.1.1. Milling

Milling can be employed to alter the inherent ultrastructure of lignocelluloses and degree of crystallinity, and consequently make it more amenable to cellulase [57]. Milling and size reduction have been applied prior to enzymatic hydrolysis, or even other pretreatment processes with dilute acid, steam or ammonia, on several lignocellulosic waste materials, MSW and activated sludge [57–60]. Among the milling processes, colloid mill, fibrillator and dissolver are suitable only for wet materials, e.g. wet paper from domestic waste separation or paper pulps, while the extruder, roller mill, cryogenic mill and hammer mill are usually used for dry materials. The ball mill can be used for either dry or wet materials. Grinding with hammer milling of waste paper is a favorable method [61].

Milling can improve susceptibility to enzymatic hydrolysis by reducing the size of the materials [62], and degree of crystallinity of lignocelluloses [25], which improves enzymatic degradation of these materials toward ethanol or biogas. Without any pretreatment, corn stover with sizes of 53–75 μm was 1.5 times more productive than larger corn stover particles of 425–710 μm [62]. Sidiras and Koukios [63] showed that due to crystallinity reduction by ball milling, saccharification of more than 50% of straw cellulose with minimal glucose degradation becomes possible at mild hydrolytic conditions. The crystallinity index of Solka Floc by ball milling changed from 74.2 to 4.9% [25]. The milling process has been studied prior to and in combination with enzymatic hydrolysis, where mechanical actions, mass transport and enzymatic hydrolysis are performed simultaneously in order to improve the hydrolysis process. The attrition mill bioreactor [64] and the intensive mass transfer reactor including ferromagnetic particles and two ferromagnetic inductors [65] are two examples of these processes.

Mais *et al*. [57] used a ball mill reactor for the pretreatment and hydrolysis of α-cellulose and SO_2_-impregnated steam-exploded Douglas fir wood chips. They reported the number of ball beads as an effective parameter to improve enzymatic hydrolysis of α-cellulose. They obtained up to 100% hydrolysis of lignocellulosic substrate with a relatively low enzyme loading (10 filter paper units/g of cellulose) when the materials were pretreated with the mills. Jim and Chen [66] studied superfine grinding, i.e. in the order of 60 μm, of steam-exploded rice straw. Rice straw was cut to 5–8 cm and steam exploded at 180, 195, 210 and 220 °C for 4–5 min separately by saturated steam. The steam-treated material was then pulverized using a vegetation disintegrator, and was put into superfine grinding using a fluidized-bed opposed jet mill. The enzymatic hydrolysis of the superfine ground straw gained the highest hydrolytic rate and yielded a very high reducing sugar.

It was shown that smaller particles were better digested in biogas production, but size reduction would have been more efficient if combined with other pretreatments. A study for improvement of biogas production from rice straw showed that a combination of grinding, heating, and ammonia treatment (2%) resulted in the highest biogas yield [59]. In another study, grinding of municipal solid waste from 2.2 to 1.1 mm had no effect on mesophilic digestion, but improved thermophilic digestion by 14% [6]. Similar work on waste-activated sludge showed a substantial improvement (16–110%) in volatile solid destruction as the effect of mechanical shear [60]. However, this effect was sludge-dependent.

Ball milling involves significant energy costs. It was suggested to use a continuous stirred tank reactor (CSTR) located between a ball mill and a hollow-fiber cartridge, in order to decrease the energy costs [67]. Another disadvantage of milling is its inability to remove the lignin which restricts the access of the enzymes to cellulose and inhibit cellulases [30, 68].

#### 4.1.2. Irradiation

Irradiation by e.g. gamma rays, electron beam and microwaves can improve enzymatic hydrolysis of lignocelluloses. The combination of the radiation and other methods such as acid treatment can further accelerate enzymatic hydrolysis [69, 70]. Irradiation has enhanced enzymatic degradation of cellulose into glucose. However, pre-irradiation is more effective in air than in acid solution [70]. Kumakura and Kaetsu [71] studied the effect of irradiation for pretreatment of bagasse prior to its enzymatic hydrolysis. The pretreated bagasse resulted in double yield of glucose by the hydrolysis compared to the untreated one. The cellulose component of the lignocellulose materials can be degraded by irradiation to fragile fibers and low molecular weight oligosaccharides and even cellobiose [71]. It could be due to preferential dissociation of the glucoside bonds of the cellulose molecular chains by irradiation in the presence of lignin. A very high irradiation, above 100 MR, can lead to the decomposition of oligosaccharides and the glucose ring structure [71]. The enzymatic hydrolysis of filter paper with no lignin was not improved by irradiation pretreatment. Furthermore, enzymatic hydrolysis of newspapers with small amounts of lignin was slightly improved by irradiation. Therefore, the effect of radiation should be correlated with the presence of lignin as well as the structure such as crystallinity and density [71–74]. However, the irradiation methods are expensive and have difficulties in industrial application.

Ultrasound is a means used for pretreatment in biogas production. It can be used for disintegration of waste-activated sludge and aquaculture effluents [75–78]. In this method, the sludge flocculi are disintegrated and the bacterial cells’ walls are disrupted [78]. Several factors such as ultrasonic density and intensity, sludge pH and sludge concentration have impact on the disintegration [79]. In addition to sonication, other methods such as cavitation, repeated freezing and defreezing, and heating at low temperatures of e.g. 60–170°C for 5–30 min or high temperatures of 180–200 °C for 10 s, can improve cell disruption and lysing [80, 81]. The other physical methods such as γ-irradiation [82], microwaves [83–85] and electrical pulses [86] have also been used to improve formation of biogas from waste materials.

### 4.2. Physico-chemical pretreatment

Pretreatments that combine both chemical and physical processes are referred to as physico-chemical processes [87]. We review the most important processes of this group in this section.

#### 4.2.1. Steam explosion (autohydrolysis)

Among the physico-chemical processes, steaming with or without explosion (autohydrolysis) has received substantial attention in pretreatment for both ethanol and biogas production. The pretreatment removes most of the hemicellulose, thus improving the enzymatic digestion. In steam explosion, the pressure is suddenly reduced and makes the materials undergo an explosive decompression. High pressure and consequently high temperature, typically between 160 and 260 °C, for a few seconds (e.g. 30 s) to several minutes (e.g. 20 min), were used in steam explosion [49, 88–99]. The steam explosion process is well documented and was tested in lab-and pilot processes by several research groups and companies. Its energy cost is relatively moderate, and it satisfies all the requirements of the pretreatment process.

The process of steam explosion was demonstrated on a commercial scale at the Masonite plants [24]. Increase in temperature up to a certain level can effectively release hemicellulosic sugars. However, the sugars loss steadily increases by further increasing the temperature, resulting in a decrease in total sugar recovery [100]. Ruiz *et al*. [100] studied steam explosion for pretreatment of sunflower stalks before enzymatic hydrolysis at a temperature in the range of 180–230 °C. The highest glucose yield was obtained in steam-pretreated sunflower stalks at 220 °C, while the highest hemicellulose recovery was obtained at 210 °C pre-treatment temperature. Using a steam explosion process for pretreatment of poplar (*Populus nigra*) biomass, at 210 °C and 4 min, resulted in cellulose recovery above 95%, enzymatic hydrolysis yield of about 60%, and 41% xylose recovery in the liquid fraction. Furthermore, the large particles can be used for poplar biomass, since no significant effect of particle size on enzymatic hydrolysis was observed [101].

Ballesteros *et al*. [92] applied steam explosion for production of ethanol from several lignocellulosic materials with *Kluyveromyces marxianus*. The poplar and eucalyptus chips were treated at 210°C for 4 min; wheat straw at 190 °C for 8 min; *Brassica carinata* residue at 210 °C at 8 min; and sweet sorghum bagasse at 210 °C for 2 min. Steam explosion extensively solubilized the hemicellulosic sugars and decreased 75–90% of xylose content, depending on the substrate. It is possible to combine steaming and mechanical treatment to effectively disrupt the cellulosic structure. Several combination technologies have been developed [24, 102, 103].

Steam explosion and thermal pretreatments are widely investigated for improving biogas production from different dedicated materials such as forest residuals [104] and wastes of e.g. activated sludge [105–108], cattle manure [109] or municipal solid wastes [110]. However, there are several investigations on combining “thermal” pretreatment with addition of bases such as NaOH, which usually give a better result than individual thermal or chemical pretreatment (e.g. [111–113]).

Special care should be taken in selecting the steam explosion conditions in order to avoid excessive degradation of the physical and chemical properties of the cellulose. In very harsh conditions, lower enzymatic digestibility of lignocelluloses may also be observed after steam explosion. For instance, generation of condensation substances between the polymers in steam explosion of wheat straw may lead to a more recalcitrant residue [114].

#### 4.2.2. Steam explosion with addition of SO_2_

Steam pretreatment can be performed with addition of sulfur dioxide (SO_2_), while the aim of adding this chemical is to improve recovering both cellulose and hemicellulose fractions. The treatment can be carried out by 1–4% SO_2_ (w/w substrate) at elevated temperatures, e.g. 160–230 °C, for a period of e.g. 10 min [53]. Eklund *et al*. [53] studied steam pretreatment of willow with the addition of SO_2_ or H_2_SO_4_ in order to recover both cellulose and hemicellulose. The maximum glucose yield, 95%, was obtained when the willow was treated with 1% SO_2_ at 200 °C. However, the yield of xylose recovery by SO_2_ was not as high as pretreatment with dilute sulfuric acid.

#### 4.2.3. Ammonia fiber explosion (AFEX)

AFEX is one of the alkaline physico-chemical pretreatment processes. Here the biomass is exposed to liquid ammonia at relatively high temperature (e.g. 90–100 °C) for a period of e.g. 30 min, followed by immediate reduction of pressure. The effective parameters in the AFEX process are ammonia loading, temperature, water loading, blowdown pressure, time, and number of treatments [46]. The AFEX process produces only a pretreated solid material, while some other pretreatments such as steam explosion produce a slurry that can be separated in a solid and a liquid fractions [115].

The AFEX process can either modify or effectively reduce the lignin fraction of the lignocellulosic materials, while the hemicellulose and cellulose fractions may remain intact. At optimum conditions, AFEX can significantly improve the enzymatic hydrolysis. The optimum conditions for AFEX depend on the lignocellulosic materials. For example, the optimum conditions in pretreatment of switchgrass were reported to be about 100°C, ammonia loading of 1:1 kg of ammonia per kg of dry matter, and 5 min retention time [43]. One of the major advantages of AFEX pretreatment is no formation of some types of inhibitory by-products, which are produced during the other pretreatment methods, such as furans in dilute-acid and steam explosion pretreatment. However, part of phenolic fragments of lignin and other cell wall extractives may remain on the cellulosic surface. Therefore, washing with water might be necessary to remove part of these inhibitory components, although increasing the amount of wastewater from the process [116]. However, there are some disadvantages in using the AFEX process compared to some other processes. AFEX is more effective on the biomass that contains less lignin, and the AFEX pretreatment does not significantly solubilize hemicellulose compared to other pretreatment processes such as dilute-acid pretreatment. Furthermore, ammonia must be recycled after the pretreatment to reduce the cost and protect the environment [23, 28, 117].

#### 4.2.4. CO_2_ explosion

Supercritical carbon dioxide has been considered as an extraction solvent for non-extractive purposes, due to several advantages such as availability at relatively low cost, non-toxicity, non-flammability, easy recovery after extraction, and environmental acceptability [118]. Supercritical carbon dioxide displays gas-like mass transfer properties, besides a liquid-like solvating power [119]. It was shown that in the presence of water, supercritical CO_2_ can efficiently improve the enzymatic digestibility of aspen (hardwood) and southern yellow pine (softwood) [120]. The delignification with carbon dioxide at high pressures can be improved by co-solvents such as ethanol–water or acetic acid–water, and can efficiently increase the lignin removal [121]. Carbon dioxide molecules should be comparable in size to those of water and ammonia, and should be able to penetrate small pores accessible to water and ammonia molecules.

Simultaneous pretreatment by CO_2_ explosion and enzymatic hydrolysis in one step has also been of interest [118, 122]. Park *et al*. [122] obtained 100% glucose yield, while applying supercritical CO_2_ and enzymatic hydrolysis of cellulose simultaneously. The cellulase was sustained at pressures of up to 160 bar for 90 min at 50 °C under supercritical carbon dioxide. They found that kinetic constants of hydrolysis under supercritical conditions were increased, compared to those under atmospheric conditions. Zheng and Tsao [118] showed cellulase enzyme to be stable in supercritical CO_2_ at a temperature of 35 °C. Only a slight decay indicates a loss of activity of about 10% after 5 days.

Explosion pretreatments of the cellulosic materials by supercritical carbon dioxide were studied by Zheng *et al*. [119]. Upon an explosive release of the carbon dioxide pressure, the disruption of the cellulosic structure should increase the accessible surface area of the substrate for enzymatic hydrolysis. Temperature is an important factor in the cellulosic hydrolysis. The experiments can be carried out at either supercritical or subcritical temperature (respectively above and below 31.1 °C). Experimental results indicated that subcritical carbon dioxide is less effective than supercritical [119]. A reason for such retardation in subcritical carbon dioxide is likely to be low diffusion in liquid carbon dioxide. In comparison with supercritical temperatures, carbon dioxide molecules at subcritical conditions find it relatively hard to penetrate the pores in the cellulosic structures, and then disrupt them when the carbon dioxide pressure is released suddenly. The higher pressure of carbon dioxide resulted in the higher glucose yield, which indicates that higher pressure is desirable for faster penetration of the carbon dioxide molecules into the cellulosic pores [119]. Apart from these advantages, the supercritical CO_2_ process might be too expensive for industrial application.

#### 4.2.5. Liquid hot-water pretreatment

Cooking of lignocellulosic materials in liquid hot water (LHW) is one of the hydrothermal pretreatment methods applied for pretreatment of lignocellulosic materials since several decades ago in e.g. pulp industries. Water under high pressure can penetrate into the biomass, hydrate cellulose, and remove hemicellulose and part of lignin. The major advantages are no addition of chemicals and no requirement of corrosion-resistant materials for hydrolysis reactors in this process. The feedstock size reduction is a highly energy-demanding operation for the huge bulk of materials on a commercial scale; there could be no need for size reduction in LHW pretreatment. In addition, the process has a much lower need of chemicals for neutralization of the produced hydrolyzate, and produces lower amounts of neutralization residues compared to many processes such as dilute-acid pretreatment. Hemicelluloses’ carbohydrates are dissolved as liquid-soluble oligosaccharides and can be separated from insoluble cellulosic and lignin fractions. LHW can enlarge the accessible and susceptible surface area of the cellulose and make it more accessible to hydrolytic enzymes [62].

Pretreatments with steam and LHW are both hydrothermal pretreatments. Higher pentosan recovery and lower formation of inhibitory components are the main advantages of LHW pretreatment compared to steam explosion. For instance, treating of de-starched corn fiber with hot water at 160 °C for 20 min dissolved 75% of the xylan [123]. At higher temperatures, e.g. 220 °C, LHW can dissolve hemicelluloses completely and remove lignin partially within 2 min with no chemicals used [124].

Xylan removal via percolation reactor, or by base addition (adjusting the pH) during the process, has been suggested to reduce the formation of inhibitors such as furfural and degradation of xylose [98]. The pH, processing temperature, and time should be controlled in order to optimize the enzymatic digestibility by LHW pretreatment [23, 115, 125, 126]. An optimized condition for LHW pretreatment of corn stover was reported to be 190 °C for 15 min, in which 90% of the cellulose conversion was observed by subsequent enzymatic hydrolysis [125]. LHW pretreatment at 160 °C and a pH above 4.0 can dissolve 50% of the fibers from corn fibers in 20 min [126]. The results showed that the pretreatment enabled the subsequent complete enzymatic hydrolysis of the remaining polysaccharides, mainly cellulose, to the corresponding monomers. The LHW pretreatment resulted in 80% soluble oligosaccharides and 20% monosaccharides with less than 1% of the carbohydrates lost to degradation products.

Laser *et al*. [98] compared the performance of LHW and steam pretreatments of sugarcane bagasse, which was subsequently used in ethanol production by SSF. They performed the treatments in a 25-l reactor at 170–230 °C for 1–46 min with 1% to 8% solids concentration. The results showed that both methods can significantly improve the hydrolysis; however, the LHW resulted in much better xylan recovery compared to steam pretreatment. Under the optimum conditions, the results of LHW pretreatment were comparable with dilute-acid pretreatment processes, besides having no requirement for acid or production of neutralization wastes. They also showed that the process favored high temperatures (above 220 °C), short residence times (less than 2 min) and low solid concentration (less than 5%).

The hot water processing removes mainly hemicellulose. A two-stage process which combines the hot water for hemicellulose removal and a treatment for delignification (e.g. ammonia treatment) was also suggested for further improvement of enzymatic hydrolysis [27, 127].

#### 4.2.6. Microwave-chemical pretreatment

The microwave/chemical pretreatment resulted in a more effective pretreatment than the conventional heating chemical pretreatment by accelerating reactions during the pretreatment process [128, 129]. Zhu *et al*. [129] examined three microwave/chemical processes for pretreatment of rice straw – microwave/alkali, microwave/acid/alkali and microwave/acid/alkali/H_2_O_2_ – for its enzymatic hydrolysis and for xylose recovery from the pretreatment liquid. They found that xylose could not be recovered during the microwave/alkali pretreatment process, but could be recovered as crystalline xylose during the microwave/acid/alkali and microwave/acid/alkali/H_2_O_2_ pretreatment. The enzymatic hydrolysis of pretreated rice straw showed that the pretreatment by microwave/acid/alkali/H_2_O_2_ had the highest hydrolysis rate and glucose content in the hydrolyzate.

### 4.3. Chemical pretreatment

#### 4.3.1. Alkaline hydrolysis

Alkali pretreatment refers to the application of alkaline solutions such as NaOH, Ca(OH)_2_ (lime) or ammonia to remove lignin and a part of the hemicellulose, and efficiently increase the accessibility of enzyme to the cellulose. The alkali pretreatment can result in a sharp increase in saccharification, with manifold yields (e.g. [130]). Pretreatment can be performed at low temperatures but with a relatively long time and high concentration of the base. For instance, when soybean straw was soaked in ammonia liquor (10%) for 24 h at room temperature, the hemicellulose and lignin decreased by 41.45% and 30.16% respectively [131]. However, alkaline pretreatment was shown to be more effective on agricultural residues than on wood materials.

Vaccarino *et al*. [132] studied the effects of SO_2_, Na_2_CO_3_, and NaOH pretreatments on the enzymatic digestibility of grape marc, and the greatest degrading effects were obtained by pretreatment with 1% NaOH solution at 120°C. Silverstein *et al*. [133] studied the effectiveness of sulfuric acid, sodium hydroxide, hydrogen peroxide, and ozone pretreatments for enzymatic conversion of cotton stalks. They found that sodium hydroxide pretreatment resulted in the highest level of delignification (65% with 2% NaOH in 90 min at 121°C) and cellulose conversion (60.8%). Zhao *et al*. [134] reported that pretreatment with NaOH could obtain a higher enzymatic conversion ratio of cellulose compared with H_2_SO_4_ pretreatment. Compared with acid or oxidative reagents, alkali treatment appears to be the most effective method in breaking the ester bonds between lignin, hemicellulose and cellulose, and avoiding fragmentation of the hemicellulose polymers [135].

The alkaline pretreatment was also used as a pretreatment method in biogas production. A pretreatment with bases such as Ca(OH)_2_ could be a solution, when high loads of e.g. lipids and phenolic compounds are subjected to the digestion. Olive mill effluent is an example of seasonal waste with low pH (about 4.3), and high lipid (ca 13 g/l) and phenolic compounds concentration (ca 8 g/l). Addition of lime and bentonite greatly improves the digestion of olive mill effluents with more than 91% removal of COD [136]. In another work, a pretreatment with ultrasound and NaHCO_3_ was reported to improve the digestibility of newsprint wastes [79]. A treatment of waste-activated sludge with 0.3 g NaOH/g volatile solids (VS) at 130°C for 5 min resulted in 40–50% solubilization of VS and more than 200% improvement in methane production compared to the control experiment [137, 138]. Furthermore, treatment of the sludge with dilute NaOH (e.g. 1.6 g/l) at room or low temperature (25–55 °C) is able to improve the VS removal by 40–90% [139, 140]. A similar treatment by 5 g/kg NaOH on municipal solid waste has also improved the formation of biogas by 35% [6].

#### 4.3.2. Alkaline peroxide

Alkaline peroxide is an effective method for pretreatment of biomass. In this method, the lignocelluloses are soaked in pH-adjusted water (e.g. to pH 11–12 using NaOH) containing H_2_O_2_ at room temperatures for a period of time (e.g. 6–24 h). The process can improve the enzymatic hydrolysis by delignification. Saha and Cotta [141] showed that by using such alkaline peroxide pretreatment, wheat straw can be converted to fermentable sugars with an excellent yield (97%) by enzymatic saccharification. In another report [142], they showed that diluted alkaline peroxide treatment (7.5% H_2_O_2_, v/v; pH 11.5; 35 °C; 24 h) is an efficient method for pretreatment of rice hulls, resulting in almost complete conversion (96%) of rice hulls to sugars after enzymatic hydrolysis. No measurable furfural and hydroxymethylfurfural (HMF) were detected in the process, which makes it more fermentable/digestable compared to e.g. in dilute-acid pretreatment. Mishima *et al*. [143] examined twenty chemical pretreatments in order to improve the efficiency of enzymatic hydrolysis of water hyacinth and water lettuce. It was shown that the alkaline/oxidative pretreatment, in which NaOH and H_2_O_2_ were used, was the most effective method for improving the enzymatic hydrolysis.

Sun *et al*. [114] studied a two-stage process based on steam explosion pretreatment followed by alkaline peroxide post-treatment. Wheat straw was first steamed at 200–220 °C and 15–22 bar. The washed fiber was then delignified by 2% H_2_O_2_ at 50 °C for 5 h under pH 11.5. The steam explosion pretreatment resulted in a significant loss in hemicelluloses, and about 11–12% lignin removal, while the alkaline peroxide post-treatment resulted in 81–88% removal of the original lignin, which altogether removed 92–99% of the original lignin from wheat straw. They also showed that the basic structure remaining is of lignin nature only, which is important for those who plan biomass strategies especially in terms of chemical uses of lignin. Curreli *et al*. [144] suggested two steps, mild alkaline/oxidative pretreatment at low temperature (25–40 °C) and low concentration of chemicals. Alkaline pretreatment (1% NaOH for 24 h) in the first step solubilizes hemicellulose, and a second alkaline/oxidative step (1% NaOH and 0.3% H_2_O_2_ for 24 h) in order to solubilize and oxidize lignin. The pretreatment is also useful in removing waxes, silica, and the waterproof cutins that coat plant tissue.

#### 4.3.3. Organosolv process

Organosolv can be used to provide treated cellulose suitable for enzymatic hydrolysis, using an organic or aqueous organic solvent to remove or decompose the network of lignin and possibly a part of the hemicellulose [144–147]. In this process, lignocellulose is mixed with organic liquid and water and heated to dissolve the lignin and part of the hemicellulose, leaving reactive cellulose in the solid phase. In addition, a catalyst may be added either to reduce the operating temperature or to enhance the delignification process [24]. Lignin in the biomass can be extracted from the solvent for e.g. generation of electricity, process heat, lignin-based adhesives and other products, due to its high purity and low molecular weight [148].

In organosolv pretreatment of lignocellulosic materials, a large number of organic or aqueous-organic solvents at temperatures of 150–200 °C can be used with or without addition of catalysts such as oxalic, salicylic, and acetylsalicylic acid. Furthermore, the solvent may accompany acetic acid released from acetyl groups developed by hydrolysis of hemicelluloses. A variety of organic solvents such as alcohols, esters, ketones, glycols, organic acids, phenols, and ethers have been used. However, the price of solvent and simplicity in recovery of solvent should also be considered. The applied solvents should be separated by e.g. evaporation and condensation, and recycled to reduce the operational costs of the process. Removal of solvents from the pretreated cellulose is usually necessary because the solvents might be inhibitors to the enzymatic hydrolysis and fermentation or digestion of hydrolyzate [28]. Araque *et al*. [149] studied the organosolv acetone–water for pretreatment. They found the highest ethanol yield to be 99.5% after pretreatment at 195 °C, 5 min, pH 2.0, and 1:1 ratio of acetone-water. For economic reasons, the use of low-molecular-weight alcohols such as ethanol and methanol has been favored over alcohols with higher boiling points, e.g. ethylene glycol, tetrahydrofurfuryl alcohol [24, 28, 41]. Ethanol is a common solvent, although it inhibits hydrolytic enzymes [23]. It should therefore be removed from the solid fraction before enzymatic hydrolysis. The main advantage of the use of solvents over other chemical pretreatments is that relatively pure, low-molecular-weight lignin is recovered as a by-product [28, 103].

Organosolv can be used together with acid hydrolysis to separate hemicellulose and lignin in a two-stage fractionation. Papatheofanous *et al*. [150] suggested such a system for pretreatment of biomass. Lignocellulosic raw material can first be treated with dilute aqueous acid (0.5–2.5 N sulfuric acid) at about 100°C for 10–60 min in order to selectively hydrolyze the hemicellulosic fraction. The aim of the second stage of the process is delignification of the pretreated lignocellulose by acidic conditions (2 N sulfuric acid) at about 81°C for 90 min. In this stage, ethanol is added (62.5–87.5%) to the system to provide the medium for dissolving and recovery of lignin generated under the acidic conditions. Negligible cellulose loss (less than 2% w/w of original cellulose) and high lignin removal (more than 70% w/w of original lignin) makes the two-stage low-temperature acid-catalyzed process interesting for laboratory pretreatment of lignocellulose before enzymatic hydrolysis.

#### 4.3.4. Wet oxidation

Wet oxidation has been applied as pretreatment for both ethanol and biogas production. In this process, the materials are treated with water and air or oxygen at temperatures above 120°C (e.g. 148–200°C) for a period of e.g. 30 min [151–153]. The temperature, followed by reaction time and oxygen pressure, are the most important parameters in wet oxidation [154]. The process is exothermic, and therefore it becomes self-supporting with respect to heat while the reaction is initiated [154]. Wet oxidation of the hemicellulose fraction is a balance between solubilization and degradation. This process is an effective method in separating the cellulosic fraction from lignin and hemicellulose [24, 155]. Oxygen participates in the degradation reactions and allows operation at comparatively reduced temperatures by enhancing generation of organic acids. However, the control of reactor temperature is critical because of the fast rates of reaction and heat generation [153]. The main reactions in wet oxidation pretreatment are the formation of acids from hydrolytic processes, as well as oxidative reactions. All three fractions of lignocellulosic materials are affected in this process. The hemicelluloses are extensively cleaved to monomeric sugars; the lignins undergo both cleavage and oxidation; and cellulose is partly degraded. The cellulose becomes highly susceptible to enzymatic hydrolysis [156].

Bjerre *et al*. [157] combined wet oxidation and alkaline hydrolysis for wheat straw pretreatment. The process resulted in a relatively highly convertible cellulose (85% conversion yield) and hemicellulose. However, addition of some alkaline agent such as sodium carbonate had only a small effect on the concentration of solubilized hemicellulose. Meanwhile, alkaline pretreatment conditions significantly decreased the degradation of hemicellulose to inhibitors, e.g. furfural [158]. Martin et al. [159] studied wet oxidation as a pretreatment method for enhancing the enzymatic convertibility of sugarcane bagasse. The pretreatment at 195 °C for 15 min solubilized 93–94% of hemicelluloses and 40–50% of lignin. The alkaline wet oxidation pretreatment at 185 °C for 5 min solubilized only 30% of hemicelluloses and 20% of lignin; meanwhile the alkaline condition reduced the formation of furans. The highest sugar yield in the liquid fraction was obtained at 185 °C, 5 min and acidic pH. The acidic wet oxidation pretreatment at 195 °C for 15 min resulted in the highest formation of carboxylic acids, phenols and furans, resulting in loss of a significant part of the polysaccharides due to degradation and formation of the by-products.

Lissens *et al*. [160] used wet oxidation to improve anaerobic biodegradability and methane yields of several raw biowastes (food waste, yard waste, and digested biowaste treated in a full-scale biogas plant). Wet oxidation temperature (185–220°C) and oxygen pressure (0–12 bar) for 15 min were used to evaluate their effect on the methane yield. The wet oxidation process reportedly increased methane yields by approximately 35–70% from raw and digested lignocellulosic biowastes.

Similar to many other delignification methods, the lignin produced by wet oxidation cannot be used as a fuel, since a major part of the lignin undergoes both cleavage and oxidation. This phenomenon considerably reduces the income from this by-product at industrial scale for ethanol production from lignocellulosic materials [161].

Wet oxidation can also be performed by oxidation agents such as hydrogen peroxide (H_2_O_2_). Azzam [162] showed that the pretreatment with hydrogen peroxide greatly enhanced the susceptibility of cane bagasse to enzymatic hydrolysis. About 50% of the lignin and most hemicellulose were solubilized by treating the biomass with 2% H_2_O_2_ at 30°C within 8 h, giving 95% efficiency of glucose production from cellulose enzymatic hydrolysis.

#### 4.3.5. Ozonolysis pretreatment

Pretreatment of lignocellulosic materials can be performed by treatment with ozone, referred to as “ozonolysis” pretreatment. This method can effectively degrade lignin and part of hemicellulose. The pretreatment is usually carried out at room temperature, and does not lead to inhibitory compounds [163]. However, ozonolysis might be expensive since a large amount of ozone is required [28]. The main parameters in ozonolysis pretreatment are moisture content of the sample, particle size, and ozone concentration in the gas flow. Among these parameters, an essential factor is the percentage of water in the feed, and it has the most significant effect on the solubilization. The optimum water content was found to be around 30%, corresponding to the saturation point of the fibers. This is an attractive pretreatment method since it does not leave acidic, basic, or toxic residues in the treated material [164].

Ozonolysis pretreatment for biogas production was investigated to improve digestion of several wastes such as sewage-activated sludge [165–167] and olive mill waste [168]. The ozone also reduced the phenolic compounds present in olive mill waste, which are toxic to methanogenic bacteria, and resulted in improvement of the digestion.

#### 4.3.6. Acid hydrolysis pretreatment

Treatment of lignocellulosic materials with acid at a high temperature can efficiently improve the enzymatic hydrolysis. Sulfuric acid is the most applied acid, while other acids such as HCl and nitric acid were also reported [3]. The acid pretreatment can operate either under a high temperature and low acid concentration (dilute-acid pretreatment) or under a low temperature and high acid concentration (concentrated-acid pretreatment). The lower operating temperature in concentrated-acid pretreatment (e.g. 40 °C) is a clear advantage compared to dilute-acid processes. However, high acid concentration (e.g. 30–70%) in the concentrated-acid process makes it extremely corrosive and dangerous. Therefore, this process requires either specialized non-metallic constructions or expensive alloys. The acid recovery, which is necessary in the concentrated-acid process for economical reasons, is an energy-demanding process. On the other hand, the neutralization process produces large amounts of gypsum. The high investment and maintenance costs also reduce the commercial interest in this process as a commercial option [23, 99, 169].

Dilute-acid hydrolysis is probably the most commonly applied method among the chemical pretreatment methods. It can be used either as a pretreatment of lignocellulose for enzymatic hydrolysis, or as the actual method of hydrolyzing to fermentable sugars. Different types of reactors such as batch, percolation, plug flow, countercurrent, and shrinking-bed reactors, for either pretreatment or hydrolysis of lignocellulosic materials by the dilute-acid processes, have been applied. These processes and different aspects of dilute-acid hydrolysis and pretreatment have recently been reviewed [3, 4]. At an elevated temperature (e.g. 140–190 °C) and low concentration of acid (e.g. 0.1–1% sulfuric acid), the dilute-acid treatment can achieve high reaction rates and significantly improve cellulose hydrolysis. Almost 100% hemicellulose removal is possible by dilute-acid pretreatment. The pretreatment is not effective in dissolving lignin, but it can disrupt lignin and increases the cellulose’s susceptibility to enzymatic hydrolysis [23, 170].

Dilute-acid pretreatment can be performed either in short retention time (e.g. 5 min) at high temperature (e.g. 180 °C) or in a relatively long retention time (e.g. 30–90 min) at lower temperatures (e.g. 120 °C). Sun and Cheng [171] pretreated rye straw and Bermuda grass for ethanol production by enzymatic hydrolysis at 121°C with different sulfuric acid concentrations (0.6, 0.9, 1.2 and 1.5%, w/w) and residence times (30, 60, and 90 min). Emmel *et al*. [15] pretreated *Eucalyptus grandis* impregnated with 0.087 and 0.175% (w/w) H_2_SO_4_ at 200–210°C for 2–5 min. The best conditions for hemicellulose recovery were obtained at 210°C for 2 min, while a lower pretreatment temperature of 200°C was enough to obtain the highest yield of cellulose conversion (90%) by enzymatic hydrolysis.

The optimum conditions for the highest hemicellulosic sugars recovery do not necessarily mean the most effective conditions for enzymatic hydrolysis. Cara *et al*. [172] reported the maximum hemi-cellulose recovery (83%) of olive tree biomass to be obtained at 170 °C and 1% sulfuric acid concentration, but the enzyme accessibility of the corresponding pretreated solid was not very high. The maximum enzymatic hydrolysis yield (76.5%) was obtained when pretreated at 210 °C with 1.4% acid concentration. The maximum total sugars, 75% of all sugars present in olive tree biomass, were obtained when the feedstock was pretreated by dilute acid at 180 °C with 1% sulfuric acid concentration. This indicates that the highest overall sugars, higher hemicellulose recovery and higher enzymatic hydrolysis yield can be achieved under respectively different conditions.

Pretreatment with acids such as acetic and nitric were also used to remove lignin from waste newsprints [173] and activated sludge [174] for biogas production. Cellulose-lignin association is considered to be the major limiting factor on long-term anaerobic digestion of newsprint. Pretreatment of bagasse and coconut fibers with HCl improved the formation of biogas from these materials by 31% and 74%, respectively [175]. Acetic acid cannot dissolve lignin even at a very high concentrations, e.g. as high as 80%, at elevated temperature (in a boiling water bath) for 30 min. In order to effectively dissolve significant amounts of lignin, nitric acid should be added. A treatment of newsprints with 30% acetic acid and 2% nitric acid resulted in removing 80% of the lignin and increasing the cellulose/lignin ratio from 1.6 to 9.9. This treatment gave improved digestion of the newsprints. The production of biogas increased by three times within 60 days incubation, from 97 ml CH_4_/g VS for the untreated newsprints to 364 ml CH_4_/gVS for the treated ones. A portion of the nitric acid might be replaced by another strong acid like hydrochloric acid; however, a longer reaction time may be required for pretreatments with lower concentrations of nitric acid [173].

Dilute-acid hydrolysis can be combined with other chemical treatments. Azzam [176] studied pretreated bagasse in a solution of ZnCl_2_ and 0.5% hydrochloric acid, heated at 145°C for 10 min, cooled and precipitated with acetone. The pretreated biomass was highly hydrolysable (yield of 93%) by cellulase.

The major drawback of some pretreatment methods, particularly at low pH is the formation of different types of inhibitors such as carboxylic acids, furans and phenolic compounds [4, 19]. These chemicals may not affect the enzymatic hydrolyses, but they usually inhibit the microbial growth and fermentation, which results in less yield and productivity of ethanol or biogas [3]. Therefore, the pretreatments at low pH should be selected properly in order to avoid or at least reduce the formation of these inhibitors.

### 4.4. Biological pretreatment

Microorganisms can also be used to treat the lignocelluloses and enhance enzymatic hydrolysis. The applied microorganisms usually degrade lignin and hemicellulose but very little part of cellulose, since cellulose is more resistance than the other parts of lignocelluloses to the biological attack. Several fungi, e.g. brown-, white- and soft-rot fungi, have been used for this purpose. White-rot fungi are among the most effective microorganisms for biological pretreatment of lignocelluloses [28].

Taniguchi *et al*. [177] evaluated biological pretreatment of rice straw using four white-rot fungi (*Phanerochaete chrysosporium, Trametes versicolor, Ceriporiopsis subvermispora,* and *Pleurotus ostreatus*) on the basis of quantitative and structural changes in the components of the pretreated rice straw as well as susceptibility to enzymatic hydrolysis. Pretreatment with *P. ostreatus* resulted in selective degradation of the lignin rather than the holocellulose component, and increased the susceptibility of rice straw to enzymatic hydrolysis. Some bacteria can also be used for biological pretreatment of lignocellulosic materials. Kurakake *et al*. [178] studied the biological pretreatment of office paper with two bacterial strains, *Sphingomonas paucimobilis* and *Bacillus circulans*, for enzymatic hydrolysis. Biological pretreatment with the combined strains improved the enzymatic hydrolysis of office paper from municipal wastes. Under optimum conditions, the sugar recovery was enhanced up to 94% for office paper.

Biological treatments with microorganisms or enzymes are also investigated to improve digestion in biogas production. The biological pretreatment might be used not only for lignin removal, but also for biological removal of specific components such as antimicrobial substances. Solid-state fermentation of orange peels by fungal strains of *Sporotrichum, Aspergillus, Fusarium and Penicillum* enhanced the availability of feed constituents and reduced the level of the antimicrobial substances [179]. In a similar work, cultivation of white-rot fungi was used to detoxify olive mill wastewater and improve its digestion [180].

Low energy requirement, no chemical requirement, and mild environmental conditions are the main advantages of biological pretreatment. However, the treatment rate is very low in most biological pretreatment processes [28].

## 5. Concluding remarks

Several pretreatment methods have been presented for lignocelluloses and waste materials in order to improve ethanol or biogas production. All these methods should make the lignocelluloses available to the enzymatic attack, where crystallinity of cellulose, its accessible surface area and protection by lignin and hemicellulose are the main factors in order to obtain an efficient hydrolysis. In addition, the efficient utilization of the hemicelluloses is an opportunity to reduce the cost of ethanol or biogas production. Diverse advantages have been reported for most of the pretreatment methods, which make them interesting for industrial applications. While methods such as dilute acid, hot water, AFEX, ammonia recycle percolation, and lime are capital-intensive [117], some other methods such as biological pretreatment are extremely slow [28]. Furthermore, some technological factors such as energy balance, solvent recycling and corrosion, as well as environmental factors such as wastewater treatment, should be carefully considered for the selected method.

## Figures and Tables

**Table 1.** Pretreatment processes of lignocellulosic materials

Pretreatment method	Processes	Studied application	Possible changes in biomass	Notable remarks	Selected References
**Physical pretreatments**	**Milling:** - *Ball milling* - *Two-roll milling* - *Hammer milling* - *Colloid illing* - *Vibro energy milling*	Ethanol	- Increase in accessible surface area and pore size - Decrease in cellulose crystallinity - Decrease in degrees of polymerization	- Most of the methods are highly energy-demanding - Most of them cannot remove the lignin - It is preferable not to use these methods for industrial applications - No chemicals are generally required for these methods	[57, 58, 63]
	**Irradiation:** - *Gamma-ray irradiation* - *Electron-beam irradiation* - *Microwave irradiation*	Ethanol and biogas			[74, 82–85]
	**Others:** - *Hydrothermal* - *High pressure steaming* - *Expansion* - *Extrusion* - *Pyrolysis*	Ethanol and biogas			[101, 153]
**Chemical and physicochemical pretreatments**	**Explosion:** - *Steam explosion* - *Ammonia fiber explosion (AFEX)* - CO_2_*explosion* - SO_2_*explosion*	Ethanol and biogas			[15, 37, 43, 46, 47, 50–54, 93, 95, 100, 118–120, 156]
	**Alkali:** - *Sodium hydroxide* - *Ammonia* - *Ammonium Sulfite*	Ethanol and biogas			[132, 127]
	**Acid:** - *Sulfuric acid* - *Hydrochloric acid* - *Phosphoric acid*	Ethanol and biogas	- Increase in accessible surface area - Partial or nearly complete delignification - Decrease in cellulose crystallinity - Decrease in degrees of polymerization - Partial or complete hydrolysis of hemicelluloses	- These methods are among the most effective and include the most promising processes for industrial applications - Usually rapid treatment rate - Typically need harsh conditions - There are chemical requirements	[3, 4, 21, 36]
	**Gas:** - *Chlorine dioxide* - *Nitrogen dioxide* - *Sulfur dioxide*	Ethanol and biogas			[56]
	**Oxidizing agents:** - *Hydrogen peroxide* - *Wet oxidation* - *Ozone*	Ethanol and biogas			[151, 154, 156, 157, 159, 162, 164–168]
	**Solvent extraction of lignin:** - *Ethanol-water extraction* - *Benzene-water extraction* - *Ethylene glycol extraction* - *Butanol-water extraction* - *Swelling agents*	Ethanol			[121]
**Biological pretreatments**	***Fungi and actinomycetes***	Ethanol and biogas	- Delignification - Reduction in degree of polymerization of cellulose - Partial hydrolysis of hemicellulose	- Low energy requirement - No chemical requirement - Mild environmental conditions - Very low treatment rate - Did not consider for commercial application	[158, 178–180]

## References

[1] Isa B, Post J, Furedy C (2004). Solid Waste Management and Recycling; Actors, Partnerships and Policies in Hyderabad, India and Nairobi, Kenya.

[2] Licht FO (2006). World ethanol markets: the outlook to 2015.

[3] Taherzadeh MJ, Karimi K (2007). Acid-based hydrolysis processes for ethanol from lignocellulosic materials: A review. BioResources.

[4] Taherzadeh MJ, Karimi K (2007). Enzymatic-based hydrolysis processes for ethanol from lignocellulosic materials: A review. BioResources.

[5] Sims R (2003). Biomass and resources bioenergy options for a cleaner environment in developed and developing countries.

[6] Ghosh S, Henry MP, Sajjad A, Mensinger MC, Arora JL (2000). Pilot-scale gasification of municipal solid wastes by high-rate and two-phase anaerobic digestion (TPAD). Water Sci. Technol.

[7] Buffiere P, Loisel D, Bernet N, Delgenes JP (2006). Towards new indicators for the prediction of solid waste anaerobic digestion properties. Water Sci. Technol.

[8] Sjöström E (1993). Wood chemistry: fundamentals and applications.

[9] Delmer DP, Amor Y (1995). Cellulose biosynthesis. Plant Cell.

[10] Morohoshi N, Hon DNS, Shiraishi N (1991). Chemical characterization of wood and its components. Wood and cellulosic chemistry.

[11] Ha MA, Apperley DC, Evans BW, Huxham IM, Jardine WG, Vietor RJ, Reis D, Vian B, Jarvis MC (1998). Fine structure in cellulose microfibrils: NMR evidence from onion and quince. Plant J.

[12] Talebnia F, Bafrani MP, Lundin M, Taherzadeh MJ (2008). Optimization study of citrus wastes saccharification by dilute acid hydrolysis. BioResources.

[13] Persson T, Matusiak M, Zacchi G, Jonsson A-S (2006). Extraction of hemicelluloses from process water from the production of masonite. Desalination.

[14] Lavarack BP, Giffin GJ, Rodman D (2002). The acid hydrolysis of sugarcane bagasse hemicellulose to produce xylose, arabinose, glucose, and other products. Biomass Bioenerg.

[15] Emmel A, Mathias AL, Wypych F, Ramos LP (2003). Fractionation of Eucalyptus grandis chips by dilute acid- catalysed steam explosion. Bioresource Technol.

[16] Ademark P, Varga A, Medve J, Harjunpaa V, Drakenberg T, Tjerneld F, Stalbrand H (1998). Softwood hemicellulose-degrading enzymes from Aspergillus niger: purification and properties of a beta-mannanase. J. Biotechnol.

[17] Mod RR, Ory RL, Morris NM, Normand FL (1981). Chemical properties and interactions of rice hemicellulose with trace minerals *in vitro*. J. Agr. Food Chem.

[18] O’Dwyer MH (1934). The hemicelluloses of the wood of English oak: The composition and properties of hemicellulose A, isolated from samples of wood dried under various conditions. Biochem. J.

[19] Taherzadeh MJ (1999). Ethanol from lignocellulose: physiological effects of inhibitors and fermentation strategies.

[20] Palmqvist E, Hahn-Hägerdal B (2000). Fermentation of lignocellulosic hydrolysates. II: Inhibitors and mechanisms of inhibition. Bioresource Technol.

[21] Torget R, Himmel ME, Grohmann K (1991). Dilute sulfuric acid pretreatment of hardwood bark. Bioresource Technol.

[22] Donghai S, Junshe S, Ping L, Yanping L (2006). Effects of different pretreatment modes on the enzymatic digestibility of corn leaf and corn stalk. Chinese J. Chem. Eng.

[23] Wyman CE (1996). Handbook on bioethanol: production and utilization.

[24] Chum HL, Douglas LJ, Feinberg DA, Schroeder HA (1985). Evaluation of pretreatments of biomass for enzymatic hydrolysis of cellulose.

[25] Fan LT, Lee Y, Beardmore DH (1980). Mechanism of the enzymatic hydrolysis of cellulose: Effects of major structural features of cellulose on enzymatic hydrolysis. Biotechnol. Bioeng.

[26] Grethelin HE (1985). The effect of pore size distribution on the rate of enzymatic hydrolysis of cellulosic substrates. Biotechnol.

[27] Kim S, Holtzapple MT (2006). Effect of structural features on enzyme digestibility of corn stover. Bioresource Technol.

[28] Sun Y, Cheng J (2002). Hydrolysis of lignocellulosic materials for ethanol production: A review. Bioresource Technol.

[29] Stone JE, Scallan AM, Donefer E, Ahlgren E, Hajny GJ, Reese ET (1969). Cellulases and their Applications.

[30] Berlin A, Balakshin M, Gilkes N, Kadla J, Maximenko V, Kubo S, Saddler J (2006). Inhibition of cellulase, xylanase and beta-glucosidase activities by softwood lignin preparations. J. Biotechnol.

[31] Ramos LP, Breuil C, Saddler JN (1992). Comparison of steam pretreatment of eucalyptus, aspen, and spruce wood chips and their enzymic hydrolysis. Appl. Biochem. Biotechnol..

[32] Mooney CA, Mansfield SD, Touhy MG, Saddler JN (1998). The effect of initial pore volume and lignin content on the enzymatic hydrolysis of softwoods. Bioresource Technol.

[33] Saha BC, Iten LB, Cotta MA, Wu YV (2005). Dilute acid pretreatment, enzymatic saccharification, and fermentation of rice hulls to ethanol. Biotechnol. Progr.

[34] Karimi K, Kheradmandinia S, Taherzadeh MJ (2006). Conversion of rice straw to sugars by dilute-acid hydrolysis. Biomass Bioenerg.

[35] Sanchez G, Pilcher L, Roslander C, Modig T, Galbe M, Liden G (2004). Dilute-acid hydrolysis for fermentation of the Bolivian straw material Paja Brava. Bioresource Technol.

[36] Schell DJ, Farmer J, Newman M, McMillan JD (2003). Dilute-sulfuric acid pretreatment of corn stover in pilot-scale reactor: Investigation of yields, kinetics, and enzymatic digestibilities of solids. Appl. Biochem. Biotechnol.

[37] Tucker MP, Kim KH, Newman MM, Nguyen QA (2003). Effects of temperature and moisture on dilute-acid steam explosion pretreatment of corn stover and cellulase enzyme digestibility. Appl. Biochem. Biotechnol.

[38] Nguyen QA, Tucker MP, Keller FA, Eddy FP (2000). Two-stage dilute-acid pretreatment of softwoods. Appl. Biochem. Biotechnol.

[39] Lee YY, Iyer P, Torget RW (1999). Dilute-acid hydrolysis of lignocellulosic biomass. Adv. Biochem. Eng. Biotechnol.

[40] Barl B, Biliaderis CG, Murray ED, Macgregor AW (1991). Combined chemical and enzymatic treatments of corn husk lignocellulosics. J. Sci. Food Agric.

[41] Arato C, Pye EK, Gjennestad G (2005). The lignol approach to biorefining of woody biomass to produce ethanol and chemicals. Appl. Biochem. Biotechnol.

[42] Sidiras D, Koukios E (2004). Simulation of acid-catalysed organosolv fractionation of wheat straw. Bioresource Technol.

[43] Alizadeh H, Teymouri F, Gilbert TI, Dale BE (2005). Pretreatment of switchgrass by ammonia fiber explosion (AFEX). Appl. Biochem. Biotechnol.

[44] Vlasenko EY, Ding H, Labavitch JM, Shoemaker SP (1997). Enzymatic hydrolysis of pretreated rice straw. Bioresource Technol.

[45] Dale BE, Leong CK, Pham TK, Esquivel VM, Rios I, Latimer VM (1996). Hydrolysis of lignocellulosics at low enzymes level: application of the afex process. Bioresource Technol.

[46] Holtzapple MT, Jun JH, Ashok G, Patibandla SL, Dale BE (1991). The ammonia freeze explosion (AFEX) process – A practical lignocellulose pretreatment. Appl. Biochem. Biotechnol.

[47] Ballesteros I, Oliva JM, Navarro AA, Gonzalez A, Carrasco J, Ballesteros M (2000). Effect of chip size on steam explosion pretreatment of softwood. Appl. Biochem. Biotechnol.

[48] Ogier JC, Ballerini D, Leygue JP, Rigal L, Pourquie J (1999). Ethanol production from lignocellulosic biomass. Oil Gas Sci. Technol.

[49] Boussaid A, Robinson J, Cai YJ, Gregg DJ, Saddler JR (1999). Fermentability of the hemicellulose-derived sugars from steam-exploded softwood (Douglas fir). Biotechnol. Bioeng.

[50] Sassner P, Galbe M, Zacchi G (2005). Steam pretreatment of Salix with and without SO2 impregnation for production of bioethanol. Appl. Biochem. Biotechnol.

[51] Ohgren K, Galbe M, Zacchi G (2005). Optimization of steam pretreatment of SO2-impregnated corn stover for fuel ethanol production. Appl. Biochem. Biotechnol.

[52] Tengborg C, Stenberg K, Galbe M, Zacchi G, Larsson S, Palmqvist E, Hahn-Hägerdal B (1998). Comparison of SO2 and H2SO4 impregnation of softwood prior to steam pretreatment on ethanol production. Appl. Biochem. Biotechnol.

[53] Eklund R, Galbe M, Zacchi G (1995). The influence of SO2 and H2SO4 impregnation of willow prior to steam pretreatment. Bioresource Technol.

[54] Stenberg K, Tengborg C, Galbe M, Zacchi G (1998). Optimisation of steam pretreatment of SO2-impregnated mixed softwoods for ethanol production. J. Chem. Technol. Biotechnol.

[55] McMillan JD, Himmel ME, Baker JO, Overend RP (1994). Pretreatment of lignocellulosic biomass. Enzymatic Conversion of Biomass for Fuels Production.

[56] Fan L, Lee Y, Gharpuray M (1982). The nature of lignocellulosics and their pretreatments for enzymatic hydrolysis. Adv. Biochem. Eng. Biotechnol.

[57] Mais U, Esteghlalian AR, Saddler JN, Mansfield SD (2002). Enhancing the enzymatic hydrolysis of cellulosic materials using simultaneous ball milling. Appl. Biochem. Biotechnol.

[58] Tassinari T, Macy C (1977). Differential speed two roll mill pretreatment of cellulosic materials for enzymatic hydrolysis. Biotechnol. Bioeng.

[59] Zhang RH, Zhang ZQ (1999). Biogasification of rice straw with an anaerobic-phased solids digester system. Bioresource Technol.

[60] Muller CD, Abu-Orf M, Novak JT (2007). Application of mechanical shear in an internal-recycle for the enhancement of mesophilic anaerobic digestion. Water Environ. Res.

[61] Walpot JI (1986). Enzymatic hydrolysis of waste paper. Conserv. Recycling.

[62] Zeng M, Mosier NS, Huang CP, Sherman DM, Ladisch MR (2007). Microscopic examination of changes of plant cell structure in corn stover due to hot water pretreatment and enzymatic hydrolysis. Biotechnol. Bioeng.

[63] Sidiras DK, Koukios EG (1989). Acid saccharification of ball-milled straw. Biomass.

[64] Ryu SK, Lee JM (1983). Bioconversion of waste cellulose by using an attrition bioreactor. Biotechnol. Bioeng.

[65] Sinitsyn AP, Gusakov AV, Davydkin IY, Davydkin VY, Protas OV (1993). A hyperefficient process for enzymatic cellulose hydrolysis in the intensive mass transfer reactor. Biotechnol. Lett.

[66] Jin S, Chen H (2006). Superfine grinding of steam-exploded rice straw and its enzymatic hydrolysis. Biochem. Eng. J.

[67] Henley RG, Yang RYK, Greenfield PF (1980). Enzymatic saccharification of cellulose in membrane reactors. Enzyme Microb. Tech.

[68] Mooney CA, Mansfield SD, Beatson RP, Saddler JN (1999). The effect of fiber characteristics on hydrolysis and cellulase accessibility to softwood substrates. Enzyme Microb. Tech.

[69] Kumakura M, Kaetsu I (1984). Pretreatment by radiation and acids of chaff and its effect on enzymatic hydrolysis of cellulose. Agr. Wastes.

[70] Mamar SAS, Hadjadj A (1990). Radiation pretreatments of cellulose materials for the enhancement of enzymatic hydrolysis. Radiat. Phys. Chem.

[71] Kumakura M, Kaetsu I (1983). Effect of radiation pretreatment of bagasse on enzymatic and acid hydrolysis. Biomass.

[72] Kumakura M, Kaetsu I (1978). Radiation-induced decomposition and enzymatic hydrolysis of cellulose. Biotechnol. Bioeng.

[73] Kumakura M, Kaetsu I (1982). Radiation degradation and the subsequent enzymatic hydrolysis of waste papers. Biotechnol. Bioeng.

[74] Kumakura M, Kojima T, Kaetsu I (1982). Pretreatment of lignocellulosic wastes by combination of irradiation and mechanical crushing. Biomass.

[75] McDermott BL, Chalmers AD, Goodwin JAS (2001). Ultrasonication as a pre-treatment method for the enhancement of the psychrophilic anaerobic digestion of aquaculture effluents. Environ. Technol.

[76] Wang QH, Kuninobu M, Ogawa HI, Kato Y (1999). Degradation of volatile fatty acids in highly efficient anaerobic digestion. Biomass Bioenerg.

[77] Cui R, Jahng D (2006). Enhanced methane production from anaerobic digestion of disintegrated and deproteinized excess sludge. Biotechnol. Lett.

[78] Chu CP, Lee DJ, Chang BV, You CS, Tay JH (2002). “Weak” ultrasonic pre-treatment on anaerobic digestion of flocculated activated biosolids. Water Res.

[79] Wang F, Wang Y, Ji M (2005). Mechanisms and kinetics models for ultrasonic waste activated sludge disintegration. J. Hazard. Mater.

[80] Dohanyos M, Zabranska J, Jenicek P (1997). Innovative technology for the improvement of the anaerobic methane fermentation. Water Sci. Technol.

[81] Wang Q, Noguchi C, Hara Y, Sharon C, Kakimoto K, Kato Y (1997). Studies on anaerobic digestion mechanism: Influence of pretreatment temperature on biodegradation of waste activated sludge. Environ. Technol.

[82] Lafitte-Trouque S, Forster CF (2002). The use of ultrasound and gamma-irradiation as pre-treatments for the anaerobic digestion of waste activated sludge at mesophilic and thermophilic temperatures. Bioresource Technol.

[83] Kennedy KJ, Thibault G, Droste RL (2007). Microwave enhanced digestion of aerobic SBR sludge. Water SA.

[84] Park B, Ahn JH, Kim J, Hwang S (2004). Use of microwave pretreatment for enhanced anaerobiosis of secondary sludge. Water Sci. Technol.

[85] Eskicioglu C, Terzian N, Kennedy KJ, Droste RL, Hamoda M (2007). Athermal microwave effects for enhancing digestibility of waste activated sludge. Water Res.

[86] Choi H, Jeong SW, Chung YJ (2006). Enhanced anaerobic gas production of waste activated sludge pretreated by pulse power technique. Bioresource Technol.

[87] Chandra R, Bura R, Mabee W, Berlin A, Pan X, Saddler J (2007). Substrate pretreatment: The key to effective enzymatic hydrolysis of lignocellulosics?. Adv. Biochem. Eng. Biotechnol.

[88] Varga E, Reczey K, Zacchi G (2004). Optimization of steam pretreatment of corn stover to enhance enzymatic digestibility. Appl. Biochem. Biotechnol.

[89] Ruiz E, Cara C, Ballesteros M, Manzanares P, Ballesteros I, Castro E (2006). Ethanol production from pretreated olive tree wood and sunflower stalks by an SSF process. Appl. Biochem. Biotechnol.

[90] Kurabi A, Berlin A, Gilkes N, Kilburn D, Bura R, Robinson J, Markov A, Skomarovsky A, Gusakov A, Okunev O, Sinitsyn A, Gregg D, Xie D, Saddler J (2005). Enzymatic hydrolysis of steam-exploded and ethanol organosolv-pretreated Douglas-Firby novel and commercial fungal cellulases. Appl. Biochem. Biotechnol.

[91] Cullis IF, Saddler JN, Mansfield SD (2004). Effect of initial moisture content and chip size on the bioconversion efficiency of softwood lignocellulosics. Biotechnol. Bioeng.

[92] Ballesteros M, Oliva JM, Negro MJ, Manzanares P, Ballesteros I (2004). Ethanol from lignocellulosic materials by a simultaneous saccharification and fermentation process (SFS) with Kluyveromyces marxianus CECT 10875. Process Biochem.

[93] Josefsson T, Lennholm H, Gellerstedt G (2002). Steam explosion of aspen wood. Characterisation of reaction products. Holzforschung.

[94] Ahring BK, Thomsen AB (2001). A method for processing lignocellulosic material.

[95] Carrasco JE, Saiz MC, Navarro A, Soriano P, Saez F, Martinez JM (1994). Effects of dilute-acid and steam explosion pretreatments on the cellulose structure and kinetics of cellulosic fraction hydrolysis by dilute acids in lignocellulosic materials. Appl. Biochem. Biotechnol.

[96] Mes-Hartree M, Saddler JN (1983). The nature of inhibitory materials present in pretreated lignocellulosic substrates which inhibit the enzymic hydrolysis of cellulose. Biotechnol. Lett.

[97] Pfeifer PA, Bonn G, Bobleter O (1984). Influence of biomass degradation products on the fermentation of glucose to ethanol by *Saccharomyces carlsbergensis* W 34. Biotechnol. Lett.

[98] Laser M, Schulman D, Allen SG, Lichwa J, Antal MJ, Lynd LR (2002). A comparison of liquid hot water and steam pretreatments of sugar cane bagasse for bioconversion to ethanol. Bioresource Technol.

[99] Sun XF, Xu F, Sun RC, Wang YX, Fowler P, Baird MS (2004). Characteristics of degraded lignins obtained from steam exploded wheat straw. Polym. Degrad. Stabil.

[100] Ruiz E, Cara C, Manzanares P, Ballesteros M, Castro E (2008). Evaluation of steam explosion pre-treatment for enzymatic hydrolysis of sunflower stalks. Enzyme Microb. Tech.

[101] Negro MJ, Manzanares P, Ballesteros I, Oliva JM, Cabanas A, Ballesteros M (2003). Hydrothermal pretreatment conditions to enhance ethanol production from poplar biomass. Appl. Biochem. Biotechnol.

[102] Mason WH (1926). Process and apparatus for disintegration of wood and the like.

[103] Katzen R, Madson PW, Monceaux DA, Lyons TP, Murtagh JE, Kelsall DR (1995). Use of cellulosic feedstocks for alcohol production. The Alcohols Textbook.

[104] Hooper RJ, Li J (1996). Summary of the factors critical to the commercial application of bioenergy technologies. Biomass Bioenerg.

[105] Ward A, Stensel HD, Ferguson JF, Ma G, Hummel S (1998). Effect of autothermal treatment on anaerobic digestion in the dual digestion process. Water Sci. Technol.

[106] Bougrier C, Delgenes JP, Carrere H (2007). Impacts of thermal pre-treatments on the semi-continuous anaerobic digestion of waste activated sludge. Biochem. Eng. J.

[107] Dereix M, Parker W, Kennedy K (2006). Steam-explosion pretreatment for enhancing anaerobic digestion of municipal wastewater sludge. Water Environ. Res.

[108] Bougrier C, Delgenes JP, Carrere H (2006). Combination of thermal treatments and anaerobic digestion to reduce sewage sludge quantity and improve biogas yield. Process Saf. Environ. Protect.

[109] Mladenovska Z, Hartmann H, Kvist T, Sales-Cruz M, Gani R, Ahring BK (2006). Thermal pretreatment of the solid fraction of manure: impact on the biogas reactor performance and microbial community. Water Sci. Technol.

[110] Solheim OE (2004). Method of and arrangement for continuous hydrolysis of organic material.

[111] Moeller-Chavez G, Gonzalez-Martinez S (2002). Two combined techniques to enhance anaerobic digestion of sludge. Water Sci. Technol.

[112] Kim J, Park C, Kim TH, Lee M, Kim S, Kim SW, Lee J (2003). Effects of various pretreatments for enhanced anaerobic digestion with waste activated sludge. J. Biosci. Bioeng.

[113] DiStefano TD, Ambulkar A (2006). Methane production and solids destruction in an anaerobic solid waste reactor due to post-reactor caustic and heat treatment. Water Sci. Technol.

[114] Sun XF, Xu F, Sun RC, Fowler P, Bairdd MS (2005). Characteristics of degraded cellulose obtained from steam-exploded wheat straw. Carbohyd. Res.

[115] Mosier N, Wyman C, Dale B, Elander R, Lee YY, Holtzapple M, Ladisch M (2005). Features of promising technologies for pretreatment of lignocellulosic biomass. Bioresource Technol.

[116] Chundawat SP, Venkatesh B, Dale BE (2007). Effect of particle size based separation of milled corn stover on AFEX pretreatment and enzymatic digestibility. Biotechnol. Bioeng.

[117] Eggeman T, Elander RT (2005). Process and economic analysis of pretreatment technologies. Bioresource Technol.

[118] Zheng Y, Tsao GT (1996). Avicel hydrolysis by cellulase enzyme in supercritical CO_2_. Biotechnol. Lett.

[119] Zheng Y, Lin H-M, Wen J, Cao N, Yu X, Tsao GT (1995). Supercritical carbon dioxide explosion as a pretreatment for cellulose hydrolysis. Biotechnol. Lett.

[120] Kim KH, Hong J (2001). Supercritical CO_2_ pretreatment of lignocellulose enhances enzymatic cellulose hydrolysis. Bioresource Technol.

[121] Pasquini D, Pimenta MTB, Ferreira LH, Curvelo AAdS (2005). Extraction of lignin from sugar cane bagasse and Pinus taeda wood chips using ethanol-water mixtures and carbon dioxide at high pressures. J. Supercrit. Fluid.

[122] Park CY, Ryu YW, Kim C (2001). Kinetics and rate of enzymatic hydrolysis of cellulose in supercritical carbon dioxide. Korean J. Chem. Eng.

[123] Dien BS, Li XL, Iten LB, Jordan DB, Nichols NN, O’Bryan PJ, Cotta MA (2006). Enzymatic saccharification of hot-water pretreated corn fiber for production of monosaccharides. Enzyme Microb. Tech.

[124] Sreenath HK, Koegel RG, Moldes AB, Jeffries TW, Straub RJ (1999). Enzymic saccharification of alfalfa fibre after liquid hot water pretreatment. Process Biochem.

[125] Mosier N, Hendrickson R, Ho N, Sedlak M, Ladisch MR (2005). Optimization of pH controlled liquid hot water pretreatment of corn stover. Bioresource Technol.

[126] Mosier NS, Hendrickson R, Brewer M, Ho N, Sedlak M, Dreshel R, Welch G, Dien BS, Aden A, Ladisch MR (2005). Industrial scale-up of pH-controlled liquid hot water pretreatment of corn fiber for fuel ethanol production. Appl. Biochem. Biotechnol.

[127] Kim TH, Lee YY (2006). Fractionation of corn stover by hot-water and aqueous ammonia treatment. Bioresource Technol.

[128] Zhu S, Wu Y, Yu Z, Liao J, Zhang Y (2005). Pretreatment by microwave/alkali of rice straw and its enzymatic hydrolysis. Process Biochem.

[129] Zhu S, Wu Y, Yu Z, Wang C, Yu F, Jin S, Ding Y, Chi R, Liao J, Zhang Y (2006). Comparison of three microwave/chemical pretreatment processes for enzymatic hydrolysis of rice straw. Biosyst. Eng.

[130] Kassim EA, El-Shahed AS (1986). Enzymatic and chemical hydrolysis of certain cellulosic materials. Agr. Wastes.

[131] Xu Z, Wang Q, Jiang Z, Yang X-X, Ji Y (2007). Enzymatic hydrolysis of pretreated soybean straw. Biomass Bioenerg.

[132] Vaccarino C, Lo Curto RB, Tripodo MM, Bellocco E, Laganfi G, Patan R (1987). Effect of SO_2_, NaOH and Na_2_CO_3_ pretreatments on the degradability and cellulase digestibility of grape marc. Biol. Waste.

[133] Silverstein RA, Chen Y, Sharma-Shivappa RR, Boyette MD, Osborne J (2007). A comparison of chemical pretreatment methods for improving saccharification of cotton stalks. Bioresource Technol.

[134] Zhao X, Zhang L, Liu D (2007). Comparative study on chemical pretreatment methods for improving enzymatic digestibility of crofton weed stem. Bioresource Technol.

[135] Gaspar M, Kalman G, Reczey K (2007). Corn fiber as a raw material for hemicellulose and ethanol production. Process Biochem.

[136] Beccari M, Majone M, Papini MP, Torrisi L (2001). Enhancement of anaerobic treatability of olive oil mill effluents by addition of Ca(OH)_2_ and bentonite without intermediate solid/liquid separation. Water Sci. Technol.

[137] Tanaka S, Kobayashi T, Kamiyama K, Bildan M (1997). Effects of thermochemical pretreatment on the anaerobic digestion of waste activated sludge. Water Sci. Technol.

[138] Tanaka S, Kamiyama K (2002). Thermochemical pretreatment in the anaerobic digestion of waste activated sludge. Water Sci. Technol.

[139] Lin JG, Ma YS, Chao AC, Huang CL (1999). BMP test on chemically pretreated sludge. Bioresource Technol.

[140] Heo NH, Park SC, Lee JS, Kang H (2003). Solubilization of waste activated sludge by alkaline pretreatment and biochemical methane potential (BMP) tests for anaerobic co-digestion of municipal organic waste. Water Sci. Technol.

[141] Saha BC, Cotta MA (2006). Ethanol production from alkaline peroxide pretreated enzymatically saccharified wheat straw. Biotechnol. Progr.

[142] Saha BC, Cotta MA (2007). Enzymatic saccharification and fermentation of alkaline peroxide pretreated rice hulls to ethanol. Enzyme Microb. Tech.

[143] Mishima D, Tateda M, Ike M, Fujita M (2006). Comparative study on chemical pretreatments to accelerate enzymatic hydrolysis of aquatic macrophyte biomass used in water purification processes. Bioresource Technol.

[144] Curreli N, Fadda MB, Rescigno A, Rinaldi AC, Soddu G, Sollai F, Vaccargiu S, Sanjust E, Rinaldi A (1997). Mild alkaline/oxidative pretreatment of wheat straw. Process Biochem.

[145] Itoh H, Wada M, Honda Y, Kuwahara M, Watanabe T (2003). Bioorganosolve pretreatments for simultaneous saccharification and fermentation of beech wood by ethanolysis and white rot fungi. J. Biotechnol.

[146] Pan X, Gilkes N, Kadla J, Pye K, Saka S, Gregg D, Ehara K, Xie D, Lam D, Saddler J (2006). Bioconversion of hybrid poplar to ethanol and co-products using an organosolv fractionation process: optimization of process yields. Biotechnol. Bioeng.

[147] Rolz C, de Arriola MC, Valladares J, de Cabrera S (1986). Effects of some physical and chemical pretreatments on the composition and enzymatic hydrolysis and digestibility of lemon grass and citronella bagasse. Agr. Wastes.

[148] Pan X, Arato C, Gilkes N, Gregg D, Mabee W, Pye K, Xiao Z, Zhang X, Saddler J (2005). Biorefining of softwoods using ethanol organosolv pulping: Preliminary evaluation of process streams for manufacture of fuel-grade ethanol and co-products. Biotechnol. Bioeng.

[149] Araque E, Parra C, Freer J, Contreras D, Rodriguez J, Mendonca R, Baeza J (2007). Evaluation of organosolv pretreatment for the conversion of Pinus radiata D. Don to ethanol. Enzyme Microb. Tech.

[150] Papatheofanous MG, Billa E, Koullas DP, Monties B, Koukios EG (1995). Two-stage acid-catalyzed fractionation of lignocellulosic biomass in aqueous ethanol systems at low temperatures. Bioresource Technol.

[151] Palonen H, Thomsen AB, Tenkanen M, Schmidt AS, Viikari L (2004). Evaluation of wet oxidation pretreatment for enzymatic hydrolysis of softwood. Appl. Biochem. Biotechnol.

[152] Varga E, Klinke HB, Reczey K, Thomsen AB (2004). High Solid Simultaneous Saccharification and Fermentation of Wet Oxidized Corn Stover to Ethanol. Biotechnol. Bioeng.

[153] Garrote G, Dominguez H, Parajo JC (1999). Hydrothermal processing of lignocellulosic materials. Holz Als Roh-und Werkst.

[154] Schmidt A, Thomsen A (1998). Optimization of wet oxidation pretreatment of wheat straw. Bioresource Technol.

[155] Saha BC (2003). Hemicellulose bioconversion. Ind. Microbiol. Biotechnol.

[156] Schultz TP, McGinnis GD, Biermann CJ (1984). Similarities and differences in pretreating woody biomass by steam explosion, wet oxidation, autohydrolysis, and rapid steam hydrolysis/continuous extraction.

[157] Bjerre AB, Olesen AB, Fernqvist T (1996). Pretreatment of wheat straw using combined wet oxidation and alkaline hydrolysis resulting in convertible cellulose and hemicellulose. Biotechnol. Bioeng.

[158] Ahring BK, Jensen K, Nielsen P, Bjerre AB, Schmidt AS (1996). Pretreatment of wheat straw and conversion of xylose and xylan to ethanol by thermophilic anaerobic bacteria. Bioresource Technol.

[159] Martin C, Klinke HB, Thomsen AB (2007). Wet oxidation as a pretreatment method for enhancing the enzymatic convertibility of sugarcane bagasse. Enzyme Microb. Tech.

[160] Lissens G, Thomsen AB, De Baere L, Verstraete W, Ahring BK (2004). Thermal wet oxidation improves anaerobic biodegradability of raw and digested biowaste. Environ. Sci. Technol.

[161] Galbe M, Zacchi G (2002). A review of the production of ethanol from softwood. Appl. Microbiol. Biotechnol.

[162] Azzam AM (1989). Pretreatment of cane bagasse with alkaline hydrogen peroxide for enzymatic hydrolysis of cellulose and ethanol fermentation. J. Environ. Sci. Heal.

[163] Vidal PF, Molinier J (1988). Ozonolysis of Lignin – Improvement of *in vitro* digestibility of poplar sawdust. Biomass.

[164] Neely WC (1984). Factors affecting the pretreatment of biomass with gaseous ozone. Biotechnol. Bioeng.

[165] Weemaes M, Grootaerd H, Simoens F, Verstraete W (2000). Anaerobic digestion of ozonized biosolids. Water Res.

[166] Goel R, Tokutomi T, Yasui H (2003). Anaerobic digestion of excess activated sludge with ozone pretreatment. Water Sci. Technol.

[167] Goel R, Tokutomi T, Yasui H, Noike T (2003). Optimal process configuration for anaerobic digestion with ozonation. Water Sci. Technol.

[168] Benitez FJ, BeltranHeredia J, Torregrosa J, Acero JL (1997). Improvement of the anaerobic biodegradation of olive mill wastewaters by prior ozonation pretreatment. Bioprocess Eng.

[169] Jones J, Semrau K (1984). Wood hydrolysis for ethanol production previous experience and the economics of selected processes. Biomass.

[170] Yang B, Wyman CE (2004). Effect of xylan and lignin removal by batch and flowthrough pretreatment on the enzymatic digestibility of corn stover cellulose. Biotechnol. Bioeng.

[171] Sun Y, Cheng JJ (2005). Dilute acid pretreatment of rye straw and bermudagrass for ethanol production. Bioresource Technol.

[172] Cara C, Ruiz E, Oliva JM, Saez F, Castro E (2007). Conversion of olive tree biomass into fermentable sugars by dilute acid pretreatment and enzymatic saccharification. Bioresource Technol.

[173] Xiao WP, Clarkson WW (1997). Acid solubilization of lignin and bioconversion of treated newsprint to methane. Biodegradation.

[174] Chen YG, Jiang S, Yuan HY, Zhou Q, Gu GW (2007). Hydrolysis and acidification of waste activated sludge at different pHs. Water Res.

[175] Kivaisi AK, Eliapenda S (1994). Pretreatment of bagasse and coconut fibres for enhanced anaerobic degradation by rumen microorganisms. Renew. Energy.

[176] Azzam AM (1987). Saccharification of bagasse cellulose pretreated with ZnCl_2_ and HCl. Biomass Bioenerg.

[177] Taniguchi M, Suzuki H, Watanabe D, Sakai K, Hoshino K, Tanaka T (2005). Evaluation of pretreatment with Pleurotus ostreatus for enzymatic hydrolysis of rice straw. J. Biosci. Bioeng.

[178] Kurakake M, Ide N, Komaki T (2007). Biological pretreatment with two bacterial strains for enzymatic hydrolysis of office paper. Curr Microbiol.

[179] Srilatha HR, Nand K, Babu KS, Madhukara K (1995). Fungal pretreatment of orange processing wastes by solid-state fermentation for improved production of methane. Process Biochem.

[180] Dhouib A, Ellouz M, Aloui F, Sayadi S (2006). Effect of bioaugmentation of activated sludge with white-rot fungi on olive mill wastewater detoxification. Lett. Appl. Microbiol.

